# Laparoscopic Outcomes after Normal Clinical and Ultrasound Findings in Young Women with Chronic Pelvic Pain: A Cross-Sectional Study

**DOI:** 10.3390/jcm9082593

**Published:** 2020-08-10

**Authors:** Nicola Tempest, Ekaterina Efstathiou, Zena Petros, Dharani K. Hapangama

**Affiliations:** 1 Liverpool Women’s Hospital NHS Foundation Trust, member of Liverpool Health Partners affiliations, Liverpool L8 7SS, UK; dharani@liv.ac.uk; 2 Department of Women’s and Children’s Health, Institute of Translational Medicine, University of Liverpool, member of Liverpool Health Partners affiliations, Liverpool L8 7SS, UK; katerinamini@hotmail.com (E.E.); zenapetros@hotmail.co.uk (Z.P.)

**Keywords:** laparoscopy, chronic pelvic pain, endometriosis, young women, investigation

## Abstract

Chronic pelvic pain (CPP) is one of the most common chronic pain problems experienced by women, with prevalence rates comparable to asthma and back pain. However, it is poorly understood and causative pathology is only seldom found. We aimed to establish prevalence of abnormal findings at diagnostic laparoscopy in young women with CPP after normal findings at clinical examination and pelvic ultrasound scan. Information was retrospectively collected on all laparoscopies undertaken on women aged 16–30 years with normal preoperative findings over a 24-month period. One-hundred-and-fifty women (mean age 25 years and BMI 24.5) were included with laparoscopic examination revealing normal anatomy in 110 (73.3%) and pathology in 40 (27.2%). Endometriosis was detected in 30 (20%); 25 (16.7%) stage 1, 2 (1.3%) stage 2, 2 (1.3%) stage 3 and 1 (0.7%) stage 4. Most laparoscopies carried out on young women with CPP and normal clinical examination and pelvic ultrasound scan showed no significant clinical stigmata of pelvic disease. Women should be fully informed of the multifactorial nature of CPP and there should be a comprehensive management pathway for these women, as proceeding with invasive laparoscopy does not provide additional benefit when investigating CPP in the context of risk, cost and effect on long-term wellbeing.

## 1. Introduction

Chronic pelvic pain (CPP) is one of the most common chronic pain problems experienced by women. It is defined as persistent pain, perceived to be originating in pelvic structures and lasting for a period of more than six months [1]. CPP can be associated with several gynaecological and non-gynaecological conditions [2,3,4]. It can be experienced as intermittent or constant pain in the lower abdomen or pelvis of a woman, not occurring exclusively with menstruation or intercourse and not associated with pregnancy [1,5,6]. Despite the high prevalence (38 per 1000) [7], a rate comparable to asthma (37 per 1000) and back pain (41 per 1000) [8], CPP is still poorly understood and causative pathology is not always found [9]. CPP, as with any chronic condition, can cause significant psychological, social and economic burden on the woman’s quality of life [1,5,7,10]. The increased prevalence of depression and anxiety observed in women with CPP is well documented across this and many other chronic pain conditions [5,10,11,12].

Although potentially a multifactorial disease, presently, CPP is not managed in a coherent, multidisciplinary manner. Consequently, specialists in different areas provide fragmentary and often-repeated investigations and management plans to women with CPP. In the UK, the management of women with CPP usually involves exclusion of infective causes and confirmation of normal pelvic anatomy by non-invasive investigations such as clinical examination, and pelvic ultrasound scan (USS), typically involving a referral to gynaecology services. This is followed by empirical treatment with hormonal therapy and or analgesia, as hormonal treatment can reduce pain in CPP sufferers without permanent or negative consequences on their subsequent fertility [13]. Many young women, either after a trial of empirical treatment, or after rejecting such treatment, undergo laparoscopic exploration of the pelvis to diagnose the existence/absence of causative factors for their CPP. The general consensus (echoed in multiple clinical guidelines [1,14,15,16,17]) would negate the requirement of such invasive surgery, solely for diagnosis when there is no delay in initiating empirical treatment or when there is no need for surgical treatment [18,19]. Furthermore, the diagnostic accuracy of laparoscopy, its risks and cost effectiveness have also been challenged [19,20]. There is a poor correlation between reported symptoms and the extent of disease found at laparoscopy in the context of endometriosis [20]. Laparoscopic findings that hypothetically may not be causing pain may lead to obligatory treatment, potentially contesting the justification of surgical assessment of the pelvis. Multiple associated functional abnormalities, outside the anatomical pelvis causing CPP, theoretically also need to be assessed and managed simultaneously. Their sequential considerations, usually starting with exclusion of gynaecological or anatomical abnormalities, and the long-term consequences of lack of preoperative psychosocial evaluation and stigmatising women with a single disease label remain unknown [21]. Surgical risks associated with laparoscopy are generally low [22,23,24], nonetheless, they merit consideration, given the potential for major (albeit rare) complications [24,25].

Considering this background, our objective was to establish the prevalence of abnormal findings at a diagnostic laparoscopy in younger women (<30 years of age) with CPP who have normal findings at clinical examination and pelvic USS.

## 2. Experimental Section

Liverpool Women’s Hospital (LWH) has approximately 80,000 gynaecology consultations per annum and is a tertiary referral centre with 12 full-time equivalent gynaecology consultants and a British Society for Gynaecological Endoscopy (BSGE) accredited endometriosis service. Approximately 7% of all referrals from general practitioners are for the complaint of CPP and these women generate the highest number of follow up appointments.

We retrospectively examined the operative findings of all women between the ages of 16 and 30 years who underwent a diagnostic laparoscopy with the indication of CPP, who had preoperative normal findings at a clinical examination performed by a gynaecologist with adequate training and at a pelvic USS, after a trial of hormones and or analgesia or declining medical treatment, over a 24-month period from December 2014 to December 2016 (*n* = 150) (see Table 1 for Inclusion/Exclusion). The diagnostic laparoscopies were performed following the SAGES diagnostic laparoscopy guidelines [26], all areas of the pelvis were visualised, photographs were taken and a 360-degree sweep was performed ensuring pathology was located and documented. We focused on a younger homogenous population of women with (known low risk of malignancy) no previous gynaecological surgeries who make up a significant proportion of the gynaecological referrals and in whom a diagnosis may have a huge life impact. No other diagnostic tests/examinations/questionnaires were completed other than documented above. All women reported CPP, which was the main indication for their diagnostic laparoscopy. Although other gynaecological symptoms such as dysmenorrhea and dyspareunia are expected to be common in this group, we did not collect information regarding these symptoms.

All paper/electronic hospital records of women who underwent a diagnostic laparoscopy in the given time period were examined by the authors who were part of the clinical team managing patients at LWH. Eligibility was checked by verifying normal clinical examination (documentation of absence of clinical exam findings consistent with any pelvic pathology such as cul de sac nodularity, immobility, pelvic masses or other pelvic pathology), pelvic USS findings documented in the hospital records and USS scan images stored in the picture archiving and communication system (PACS). Surgical findings at laparoscopy were obtained from the operation notes recorded by the surgeon at the time of laparoscopy and by the authors re-examining the accompanying surgical photographs surveying the upper abdomen, appendix and bowel in addition to the pelvis. The verified final data was transcribed onto a paper based proforma prior to transfer onto an excel spreadsheet for analysis, without collecting any patient identifiable information. This observational cross-sectional study did not assess the influence of a single risk factor or intervention; therefore, a conventional power calculation was not undertaken. This information gathering, as part of assessing the standard of care at LWH, was approved by the directorate audit committee and did not require ethical approval from the adult research ethics committee.

The following demographic variables were collected; woman’s age, body mass index (BMI), age at menarche, parity, hormonal contraceptive use, past medical and surgical history and if medical treatment was accepted pre-surgery. The primary, clinically significant outcome collected was the presence/absence of pelvic pathology (including the presence/ absence of endometriosis) that could potentially relate to the symptoms of CPP at diagnostic laparoscopy.

## 3. Results

One-hundred-and-fifty consecutive women aged 16 to 30 years (mean age 25 years) with CPP and confirmed normal findings at clinical and USS examination of the pelvis, who subsequently underwent a diagnostic laparoscopy, were included in this study. The mean BMI of the patient cohort was 24.5 and mean age at menarche was 12 years; 102 (68%) were nulliparous, 33 (22%) were para one, 10 (6.7%) were para two and 5 (3.3%) were para three (Table 2).

**Figure 1:**
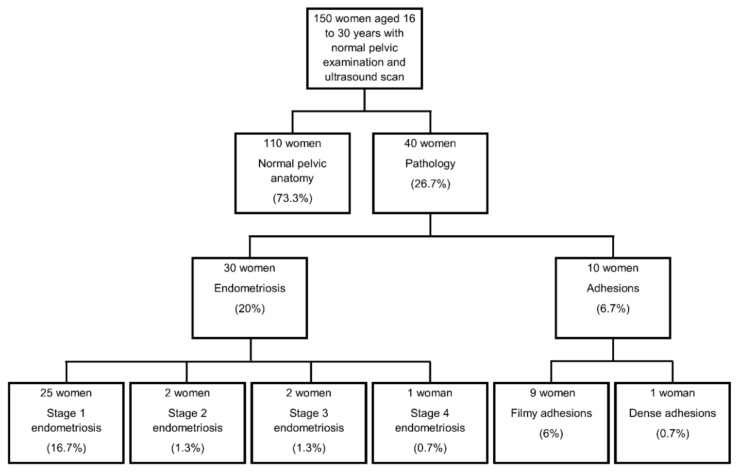
Flow chart of laparoscopy findings.

Normal pelvic anatomy without any pathology was the operative finding at diagnostic laparoscopy in 110 (73.3%) women. Abnormalities were detected in the remaining 40 (26.7%), which included endometriosis in 30 (20%), filmy adhesions in 9 (6%) and dense adhesions in 1 (0.7%) (Figure 1).

Among the women who were diagnosed with endometriosis, 25 (16.7%) had stage 1 endometriosis, 2 (1.3%) had stage 2 endometriosis, 2 (1.3%) had stage 3 endometriosis and 1 (0.7%) had stage 4 endometriosis.

Age, BMI and age at menarche were comparable between the women with and without endometriosis. An apparently higher proportion of women were nulliparous in the endometriosis group (83.3% vs. 64.2%) (Table 2).

The most commonly reported past medical history in the women diagnosed with endometriosis was depression (6 women affected (20%), compared with only 10 (8.3%) women in the non-endometriosis group). Consequently, antidepressants were the most common concurrent medication taken by women diagnosed with endometriosis, 6 women (20%), compared with only 8 women (6.7%) taking regular antidepressants in the group without endometriosis. The most commonly recorded past medical history in the women without endometriosis was irritable bowel syndrome (IBS) (14 women (11.7%) compared with 5 women (16.7%) reporting IBS diagnosis in the endometriosis group) (Table 3).

An apparently more frequent analgesic use was reported by the CPP suffers who did not have endometriosis (51 women (42%) compared with 11 women (36.7%) diagnosed with endometriosis) (Table 3).

Prior to surgery, for contraception/treatment of pelvic pain, young women in this CPP cohort who were subsequently diagnosed with endometriosis used COCP slightly more frequently than the women not found to have endometriosis (14 (46.7%) vs. 38 (31.7%)). The use of all other forms of hormonal treatment/contraception was comparable in both groups (Table 3).

According to the hospital records, clinicians offered a trial of hormonal medications prior to diagnostic laparoscopy when seen in clinic, but these were declined by 11 women (36.7%) in the group diagnosed with endometriosis and by 41 women (34.2%) in the group without endometriosis.

## 4. Discussion

Laparoscopies when normal pelvic anatomy was observed upon pelvic examination (clinical examination by a trained gynaecologist and on pelvic USS) in young women with CPP merely reconfirmed a normal pelvis with no significant clinical stigmata of pelvic disease in most. Just 20% of young women in this group were diagnosed with endometriosis, a figure similar to the background population incidence. Importantly, out of those diagnosed with endometriosis, only 3 women (2%) had advanced stage (American Fertility Society (AFS) stage 3/4). Previous studies assessing the incidence of endometriosis particularly considered either adolescents/younger women (<21 years) or older (>30 years) women. Our data therefore fills the void in the current literature providing information on the incidence of endometriosis in younger women up to 30 years of age (the most fertile female population, who have a lower incidence of sinister pathology). The benefit versus risk of conducting a diagnostic laparoscopy in this well-characterised patient population (normal clinical and USS findings obtained in a specialist setting by highly qualified practitioners) we focused on is unclear. There is considerable doubt that endometriosis is solely responsible for CPP and if surgical excision of the superficial peritoneal lesions (usually stage 1/2 disease) result in their long-term wellbeing or lasting symptom control [19]. Establishing whether treating isolated peritoneal endometriosis by surgical means is cost effective or not is important, as this forms a large part of the workload in general gynaecology, and uses a considerable amount of resources [13,19]. The frequency of analgesic use in women with/without endometriosis in our cohort concords with previous reports of women with endometriosis having the same severity of CPP as women without endometriosis [27]. Some women with endometriosis may not complain of CPP at all [8,28,29]. Other chronic pain conditions such as IBS or painful bladder syndrome may be responsible for CPP [30] despite the presence or absence of endometriosis, and women with and without endometriosis in our cohort had a similar incidence of IBS. The much higher rate (double) of depression and higher rate of anxiety reported by women with endometriosis than those women who did not have endometriosis in our cohort also confirm previous reports [12,31,32,33]. These observations highlight the complexities and the possible contributions from non-gynaecological factors in the pathophysiology of CPP [34,35]. Our data propose that simultaneous assessment and treatment of all these complaints in younger CPP suffers before resorting to invasive investigations (i.e., laparoscopy). However, this cohort of women was not followed up to assess their subsequent management or response to treatment.

Some pathology can be diagnosed with surgical visualisation of the pelvis with laparoscopy, and the National Institute for Health and Care Excellence (NICE) guideline in the UK states that delays in diagnosis of endometriosis can affect quality of life and result in disease progression, increased personal suffering, prolonged ill health and a disease state that is more difficult to treat [13]. However, there is a lack of evidence to support this statement and recommendation. The subsequent progression of endometriosis beyond the initial diagnosis remains a controversial issue with hitherto unknown natural history of the disease. There is a dearth of reliable evidence to suggest delay in diagnosis or early medical intervention may affect the long-term clinical outcomes of endometriosis. There are some studies suggesting higher rates of recurrent disease in younger women after surgical treatment, when compared with older women [36,37,38], thus future well-designed, prospective, large-scale studies with long-term follow-up are required to assess the benefit of surgical intervention for endometriosis, particularly in younger women.

As diagnostic laparoscopy has not provided any additional clinically useful information in more than 73% of women in our cohort, it could be deemed as an unnecessary invasive procedure in a large proportion of young women. Further studies are therefore warranted to assess the need, safety and cost benefit for the routine use of laparoscopy from the women’s perspective as well as from direct clinical, service provision aspects. It is thus important that women are fully counselled before surgery regarding the possibility of a “negative” laparoscopy finding and an alternative management plan is proposed for them if no gynaecological pathology is identified. Consequences of a negative laparoscopy have not been thoroughly examined. Many women with CPP after a negative laparoscopy report feeling “let down”, and they perceive their doctor may assume that “the problem is all in [their] head” [39]. The potential unfavourable effect of laparoscopy needs to be considered, since pain, in these women can be due to other overlooked causes (e.g., IBS, bladder pain syndrome, musculoskeletal disorders and somatic symptom disorder). It is proposed that the treatment for these other conditions may be delayed by invasive diagnostic laparoscopy and time lapse in referral to other clinicians. Our study provides valuable information regarding the chances of abnormal findings at diagnostic laparoscopy in young women when normal pelvic USS and clinical examination are present, to improve the preoperative counselling process. This is particularly true, with a high-quality imaging approach, when specialist sonographers survey the pelvis according to the International Deep Endometriosis Analysis group guidelines to identify deep infiltrating endometriosis [40].

The European Society of Human Reproduction and Embryology (ESHRE) guidelines recommend that in the absence of signs of deep infiltrating or ovarian endometriosis in clinical exam and imaging, a laparoscopy should not be performed purely to find/treat peritoneal disease, especially in adolescents and young adults. The NICE guidelines in the UK recommends a more modest 3-month trial of medical treatment prior to laparoscopy and echoes, like the ESHRE guidelines that lack of histology at laparoscopy does not rule out a diagnosis. Advice differs when infertility is considered, as treatment of mild/minimal endometriosis leads to a higher natural conception rate when compared with diagnostic laparoscopy alone [15]. This advice comes with the caveat that in women with unexplained infertility, diagnostic laparoscopy should not be performed just for the indication of potentially diagnosing and excising endometriosis. We present consecutive, contemporaneous data from a single, large specialist gynaecological unit. Although the collection process was retrospective in nature, our comprehensive data collection ensured accuracy of the data by consulting multiple data sources, including the paper notes, PACS images and computerised hospital notes. In our centre, the laparoscopies are conducted by experienced gynaecologists affiliated with the British Society of Gynaecological Endoscopy (BSGE) accredited endometriosis service, and our specialist gynaecological sonographers follow standardised protocols to diagnose endometriosis and other pelvic pathology. Therefore, we cannot confirm the generalisability of our data in other settings.

In our study, we took a pragmatic approach to assess outcomes following an invasive diagnostic approach for CPP patient management in an NHS gynaecological unit. Therefore, we cannot evaluate the role of associated important symptomology, such as dysmenorrhea, dyspareunia and dyschezia, that these women were experiencing as part of their CPP. However, the contribution of these (for example, dysmenorrhea, is a very common symptom [41]), may account for analgesic use, independent of endometriosis. Future studies developing patient management pathways should focus not only on gynaecological symptoms, but assessing all relevant pain symptoms comprehensively prior to embarking on invasive investigations.

## 5. Conclusions

Available evidence for CPP suggests only limited benefit of surgical treatment for mild endometriosis or filmy adhesions, yet they were the main findings in our selective cohort of young women after normal pelvic anatomy was already observed upon pelvic examination and USS. We therefore propose that a CPP management pathway in this population should incorporate empirical medical treatment, assessment and management of other associated causes of CPP, reassurance, education and supported self-management strategies [1,36,42], followed by invasive investigations as a later non-compulsory option. Further studies are urgently needed to assess the additional long-term benefit of early invasive diagnostic procedures in young women suffering with CPP over empirical, integrated, non-surgical management of CPP.

## Figures and Tables

**Table 1:** Inclusion and exclusion criteria for a cross-sectional study of patients with chronic pelvic pain (CPP) attending the LWH.

Inclusion Criteria	Exclusion Criteria
Aged 16–30 years	Aged < 16 years or > 30 years
No previous gynaecological surgery	Previous gynaecological surgery
Chronic pelvic pain	No chronic pelvic pain
Normal pelvic clinical examination	Pathology at pelvic clinical examination
Normal pelvic USS	Pathology on pelvic USS
Non-pregnant	Pregnant

**Table 2:** Demographics.

Demographics	All Women (150)	Endometriosis at Laparoscopy (30)	No Endometriosis at Laparoscopy (120)
Age, mean (SD)	24.5 (3.8)	23.5 (4.4)	23.4 (3.6)
BMI, mean (SD)	24.5 (4.1)	25 (4.0)	24.4 (4.1)
Age at menarche, mean (SD)	12.3 (1.5)	12.5 (1)	12.3 (1.5)
Nulliparous, n (%)	102 (68%)	25 (83.3%)	77 (64.2%)
Irritable bowel syndrome, n (%)	19 (12.7%)	5 (16.7%)	14 (11.7%)
Anxiety, n (%)	16 (10.7%)	4 (13.3%)	12 (10%)
Depression, n (%)	16 (10.7%)	6 (20%)	10 (8.3%)
Asthma, n (%)	12 (8%)	4 (13.3%)	8 (6.7%)
Heartburn, n (%)	11 (7.3%)	3 (10%)	8 (6.7%)
Migraine, n (%)	6 (4%)	1 (3.3%)	5 (4.2%)
PCOS, n (%)	6 (4%)	0 (0%)	6 (5%)
Ruptured ovarian cyst, n (%)	3 (2%)	1 (3.3%)	2 (1.7%)
IDDM, n (%)	2 (1.3%)	0 (0%)	2 (1.7%)
Anaemia, n (%)	2 (1.3%)	0 (0%)	2 (1.7%)
Eczema, n (%)	2 (1.3%)	1 (3.3%)	1 (0.8%)
Vit B12 deficiency, n (%)	1 (0.7%)	0 (0%)	1 (0.8%)
Haemochromatosis, n (%)	1 (0.7%)	0 (0%)	1 (0.8%)
Juvenille arthritis, n (%)	1 (0.7%)	0 (0%)	1 (0.8%)
Deaf, n (%)	1 (0.7%)	0 (0%)	1 (0.8%)
Hypermobility, n (%)	1 (0.7%)	0 (0%)	1 (0.8%)
Fibromyalgia, n (%)	1 (0.7%)	0 (0%)	1 (0.8%)
Diverticulitis, n (%)	1 (0.7%)	0 (0%)	1 (0.8%)
Psoriasis, n (%)	1 (0.7%)	0 (0%)	1 (0.8%)
Vaginismus, n (%)	1 (0.7%)	1 (3.3%)	0 (0%)
Lactose intolerance, n (%)	1 (0.7%)	0 (0%)	1 (0.8%)
Hypothyroid, n (%)	1 (0.7%)	0 (0%)	1 (0.8%)
Appendiectomy, n (%)	7 (4.7%)	0 (0%)	7 (5.8%)
Tonsilectomy, n (%)	5 (3.3%)	0 (0%)	5 (4.2%)
Gromit and adenoids, n (%)	2 (1.3%)	1 (3.3%)	1 (0.8%)
Breast augmentation, n (%)	2 (1.3%)	0 (0%)	2 (1.7%)
Breast reduction, n (%)	2 (1.3%)	1 (3.3%)	1 (0.8%)
Rhinoplasty, n (%)	1 (0.7%)	1 (3.3%)	0 (0%)
Arthroscopy, n (%)	1 (0.7%)	0 (0%)	1 (0.8%)
Kidney stone removal, n (%)	1 (0.7%)	0 (0%)	1 (0.8%)

**Table 3:** Current medication/contraceptive use.

Current Medication/Contraceptive Use	All Women (150)	Endometriosis at Laparoscopy (30)	No Endometriosis at Laparoscopy (120)
Analgesia	62 (41.3%)	11 (36.7%)	51 (42.5%)
Antidepressants	14 (9.3%)	6 (20%)	8 (6.7%)
Anti-anxiety	9 (6%)	2 (6.7%)	7 (5.8%)
Inhalers	7 (4.7%)	4 (13.3%)	3 (2.5%)
Antacids	7 (4.7%)	3 (10%)	4 (3.3%)
IBS treatment	7 (4.7%)	1 (3.3%)	6 (5%)
Laxatives	3 (2%)	1 (3.3%)	2 (1.7%)
Insulin	2 (1.3%)	0 (0%)	2 (1.7%)
Folic acid	1 (0.7%)	0 (0%)	1 (0.8%)
Vitamin B12	1 (0.7%)	0 (0%)	1 (0.8%)
Levothyroxine	1 (0.7%)	0 (0%)	1 (0.8%)
Iron	1 (0.7%)	0 (0%)	1 (0.8%)
COCP	52 (34.7%)	14 (46.7%)	38 (31.7%)
POP	20 (13.3%)	4 (13.3%)	16 (13.3%)
Depo	10 (6.7%)	2 (6.7%)	8 (6.7%)
Implant	10 (6.7%)	2 (6.7%)	8 (6.7%)
Mirena	6 (4%)	1 (3%)	5 (4.2%)
Cu coil	2 (1.3%)	0 (0%)	2 (1.7%)
